# The relationship between the ratio of gamma-glutamyltransferase to high-density lipoprotein cholesterol and the risk of diabetes mellitus using publicly available data: a secondary analysis based on a longitudinal study in Japan

**DOI:** 10.1186/s12944-023-01772-9

**Published:** 2023-01-17

**Authors:** Yue Zhao, Xing Xin, Xiao-ping Luo

**Affiliations:** 1 Department of Pediatrics, Tongji Hospital, Tongji Medical College, Huazhong University of Science and Technology, Wuhan, China; 2 Department of Obstetrics and Gynecology, Tongji Hospital, Tongji Medical College, Huazhong University of Science and Technology, Wuhan, China

**Keywords:** Ratio of gamma-glutamyltransferase to high-density lipoprotein cholesterol, Incidence of T2DM, Nonlinear relationship, Threshold effect

## Abstract

**Background:**

The ratio of gamma-glutamyltransferase to high-density lipoprotein cholesterol (GGT/HDL-C) has been highlighted in nonalcoholic fatty liver disease (NAFLD) by previous studies. However, there have been fewer investigations into the correlation between the GGT/HDL-C ratio and type 2 diabetes mellitus (T2DM) incidence. Our secondary analysis used published data from a Japanese population and aimed to investigate the role of the GGT/HDL-C ratio in the incidence of T2DM.

**Methods:**

The research was a longitudinal cohort study completed by Okamura, Takuro et al. We obtained the data from the DATADRYAD website and used it for secondary analysis only. The participants recruited from a medical program called the NAGALA database received regular medical examinations and standardized questionnaires to obtain the baseline variables. Abdominal ultrasound was used to diagnose fatty liver disease. The participants were followed up, and the duration and occurrence of T2DM were documented. The GGT/HDL-C ratio evaluated at baseline served as the independent variable, while the occurrence of diabetes served as the dependent variable.

**Results:**

A total of 15,453 cases (8,419 men and 7,034 women) were included in our study. After adjusting for age, sex, BMI, DBP, SBP, ALT, AST, TG, TC, HbA1C, FPG, drinking status, smoking status, exercise status, and fatty liver, we observed that the GGT/HDL-C ratio was positively associated with the incidence of T2DM (hazard ratio = 1.005, 95% confidence interval: 1.000 to 1.010, *P* = 0.0667). The results were consistent when the GGT/HDL-C quartile was used as a categorical variable (*P* for trend < 0.00396). A curvilinear relationship with a threshold effect was identified between the GGT/HDL-C ratio and the risk of incident T2DM. On the left of the point, a one-unit increase in the GGT/HDL-C ratio was associated with a 1.5-fold increase in the risk of incident T2DM (hazard ratio 2.57, 95% confidence interval 1.20 to 5.49). On the right of the point, when GGT/HDL-C was greater than 6.53, their relationship became saturated.

**Conclusion:**

The GGT/HDL-C ratio correlated with the incidence of T2DM in a curvilinear form with a threshold effect. Their positive relationship could be observed when GGT/HDL-C was less than 6.53.

**Supplementary Information:**

The online version contains supplementary material available at 10.1186/s12944-023-01772-9.

## Background

Type 2 diabetes mellitus (T2DM) is an epidemic metabolic disorder with a rapidly increasing incidence worldwide. The disease can lead to subsequent metabolic disorders and other complications that are hazardous to health and potentially lethal, increasing mortality.

Abnormal glucose and lipid metabolism often accompany the onset and development of T2DM [1]. The liver is the leading site in charge of lipid metabolism, glucose metabolism, and homeostasis. In clinical practice, we often use lipid indicators such as triglyceride (TG), total cholesterol (TC), HDL-C, and files of liver enzyme indicators such as ALT (alanine aminotransferase), AST (aspartate aminotransferase) and GGT (gamma-glutamyltransferase) to reflect lipid metabolism and liver function. Single and complex liver enzymes, as well as the lipid index, have been explored in research on risk factors for diabetes [2–5]. Previous studies have reported that TG/HDL-C, ALT/AST, and GGT all have associations with the incidence of T2DM, and some of them have predictive value for the incidence of T2DM [6]. Recently, the role of the GGT/HDL-C ratio in NAFLD patients has been discussed. The authors suggested that this GGT/HDL-C ratio might predict the risk of NAFLD and metabolic diseases such as diabetes [7]. To date, very little attention has been given to the role of GGT/HDL-C in patients with T2DM. To determine whether there is a link between GGT/HDL-C and the incidence of T2DM or whether GGT/HDL-C is a more comprehensive indicator for assessing the risk of T2DM, we used published data on the Japanese population to conduct secondary data analysis. This secondary analysis used the GGT/HDL-C ratio as the independent variable and the incidence of T2DM as the dependent variable to probe into the independent association.

## Methods

### Data source

We downloaded the data for this study from www.Datadryad.org, the ‘DATADRYAD’ website. According to the website's request for data usage, the Dryad data package ([8] Dryad data package: Okamura, Takuro et al. (2019), Data from Ectopic fat obesity presents the greatest risk for incident T2DM: a population-based longitudinal study, Dryad, Dataset, https://doi.org/10.5061/ dryad. 8q0p192) need to be cited when using these data.

### Study population

The longitudinal study was based on the medical examination program NAGALA (NAfld in the Gifu Area, Longitudinal Analysis). The database comes from the medical examination program at Murakami Memorial Hospital (Gifu, Japan), founded in 1994. The objective of this project is to investigate risk factors for chronic diseases. Takuro Okamura et al. [8] extracted cases from 2004 to 2015 who participated in the medical examination program at Murakami Memorial Hospital in their analysis.

The study initially enrolled 20,944 participants (12,498 male and 8,446 female), of which 863 participants (504 male and 359 female) were excluded because of missing data. Another 416 participants (278 male and 138 female) were excluded due to known liver disease; 739 participants (635 male and 104 female) were excluded due to ethanol consumption over the level regarded as toxic to the liver (420 g/w for male, 280 g/w for female) [9]; 2,321 participants (1,709 male and 612 female) were also excluded due to any medication usage at baseline; 322 patients (265 male and 58 female) and 128 patients (677 male and 131 female) were excluded due to diabetes at the baseline examination or FPG ≥ 6.1 mmol/L [10], respectively. Another 11 participants (11 male) were excluded due to having an invalid GGT/HDL-C ratio. Thus, 15,453 subjects (7,034 female and 8,419 male) remained to be part of this study.

The ethics committee of Murakami Memorial Hospital approved this study. Takuro Okamura et al. [8] obtained written informed consent for data usage from each participant.

### The data collection and measurement procedure

All the baseline variables included in the database were collected at baseline by a regular medical exam, including basic information such as age, sex, BMI (body mass index), DBP (diastolic blood pressure), SBP (systolic blood pressure), and biochemical indicators ALT, AST, GGT, TG, TC, HDL-C, glycosylated hemoglobin (HbA1C), and fasting plasma glucose (FPG). Other covariates related to lifestyle habits, such as smoking and drinking status, exercise status, and ethanol consumption, were obtained by questionnaire. B-ultrasonography was used to diagnose fatty liver disease by trained technicians. A follow-up study was performed on participants’ incidence of T2DM [11].

BMI is obtained by dividing weight (in kg) by the square of height (in meters). Okamura et al. [8] applied a standardized self-administered questionnaire to ascertain the participants’ information on medication usage, drinking, smoking, and exercise habits. We estimated average ethanol intake per week by the type and amount of alcohol consumed weekly during the prior month, and we divided ethanol consumption into four groups: 1) no or minimal, 2) light, 3) moderate, and 4) heavy; the corresponding ethanol consumption was < 40 g/week, 40 to 140 g/week, 140 to 280 g/week, and > 280 g/week [12]. Smoking status was divided into three categories: never, ex-smoker, or current smoker. Regular exercise habits were defined as participating in any sport regularly, more than once a week [13, 14]. Trained technicians diagnosed fatty liver disease by the criteria of the scoring system of abdominal ultrasonography [15]. The technicians were not informed of other personal information of the specific participants. Finally, T2DM was diagnosed by FPG ≥ 7 mmol/L, HbA1c ≥ 6.5% [16] or by self-report, and the date of diagnosis was recorded.

### Statistical analyses

Data management and analysis were performed using the statistical software package R (http://www. R-project.org, The R Foundation) and EmpowerStats (http://www.empowerstats.com, X&Y Solutions, Inc., Boston, MA). *P* < 0.05 (two-sided) was considered statistically significant.

We carried out the data analysis according to the following procedures: For the data of baseline characteristics of participants (the GGT/HDL-C was grouped in quartiles), data were presented as the means ± standard deviations for normal distribution, medians (quartiles) for skewed distribution, frequency or percentage for categorical variables. The significant differences among the GGT/HDL-C quartiles were tested with one-way ANOVA for continuous variables with a normal distribution, the Kruskal‒Wallis H test for continuous variables with a skewed distribution, and the chi-square test for categorical variables. We used Cox proportional hazard regression models to explore the predictive effect of GGT/HDL-C on the risk of T2DM. The hazard ratio (HR) and 95% confidence interval (CI) were calculated. To satisfy the recommendations comprising the STROBE statement [17], if the covariance added to the model altered the matched effect size by at least ten percent, they were adjusted [18], and we presented the results of the unadjusted model and models with different covariates that were adjusted simultaneously. For trend testing, we converted the GGT/HDL-C continuous variables into categorical variables, and the P for trend was calculated. Next, considering possible nonlinear relationships that might exist between dependent and independent variables, a generalized additive model (GAM) was applied. In terms of the smoothing plot, we used a two-piecewise linear regression model to calculate the inflection point of GGT/HDL-C on the incidence of T2DM. The recursive method automatically calculated the inflection point by using the maximum model likelihood when the inflection point appeared in the curve. In the next step, subgroup analysis was also explored by stratified Cox regression models, and we used the likelihood ratio test to test for interactions across subgroups. We used the Kaplan–Meier method to compare cumulative diabetes event rates within specific time intervals. The log-rank test compared HR and corresponding 95% CI for the events of T2DM in each GGT/HDL-C quartile.

## Results

### Baseline characteristics of participants

The baseline characteristics of the patients divided by GGT/HDL-C quartiles are shown in Table 1. The average age of the participants was 43.71 ± 8.90 years. The average GGT/HDL-C was 10.78 (1.57–342.82). The factors of age, sex, BMI, ALT, AST, TC, TG, SBP, DBP, FPG, HbA1C, and ethanol consumption were compared across the GGT/HDL-C quartiles. In the higher GGT/HDL-C group (Q3, Q4), males accounted for the majority, and relatively more participants in Q3 and Q4 were diagnosed with fatty liver disease and were current smokers compared with the lower GGT/HDL-C groups (Q1, Q2). The participants of the Q3 and Q4 groups also had a relatively higher ethanol consumption compared with the lower GGT/HDL-C group (Q1 and Q2).

**Table 1:** Baseline characteristics of participants

GGT/HDL-C ratio quartiles	Q1 (1.57–7.20)	Q2 (7.20–10.77)	Q3 (10.78–17.95)	Q4 (17.96–342.82)	*P* value
N	3862	3861	3866	3864	
Sex					< 0.001
Male	441 (11.42%)	1576 (40.82%)	2881 (74.52%)	3521 (91.12%)	
Female	3421 (88.58%)	2285 (59.18%)	985 (25.48%)	343 (8.88%)	
Age (years)	42.31 ± 8.36	43.12 ± 9.05	44.31 ± 9.25	45.09 ± 8.65	< 0.001
BMI (kg/m2)	20.36 ± 2.31	21.26 ± 2.64	22.56 ± 2.95	24.28 ± 3.08	< 0.001
SBP (mmHg)	107.48 ± 13.14	111.86 ± 13.90	116.78 ± 14.32	121.85 ± 14.50	< 0.001
DBP (mmHg)	66.40 ± 9.16	69.54 ± 9.52	73.23 ± 10.02	77.15 ± 10.09	< 0.001
ALT (IU/L)	13.00 (10.00–15.00)	15.00 (12.00–18.00)	18.00 (15.00–23.00)	26.00 (20.00–36.00)	< 0.001
AST (IU/L)	15.00 (13.00–18.00)	16.00 (13.00–19.00)	18.00 (15.00–21.00)	21.00 (17.00–26.00)	< 0.001
TC (mmol/L)	5.02 (4.47–5.61)	4.94 (4.40–5.51)	5.04 (4.47–5.66)	5.33 (4.76–5.87)	< 0.001
TG (mmol/L)	0.51 (0.38–0.69)	0.61 (0.44–0.86)	0.84 (0.60–1.20)	1.23 (0.86–1.80)	< 0.001
HbA1C (mmol/mol)	32.24 (30.06–35.52)	32.24 (30.52–35.52)	33.34 (31.15–35.52)	33.34 (31.15–36.09)	< 0.001
FPG (mg/dL)	89.00 (85.00–94.00)	91.00 (87.00–96.00)	94.00 (90.00–99.00)	97.00 (92.00–102.00)	< 0.001
Ethanol consumption (g/week)	1.00 (0.00–12.00)	1.00 (0.00–36.00)	10.27 (0.00–84.00)	36.00 (1.00–132.00)	< 0.001
Fatty liver					< 0.001
No	3777 (97.80%)	3600 (93.24%)	3123 (80.78%)	2216 (57.35%)	
Yes	85 (2.20%)	261 (6.76%)	743 (19.22%)	1648 (42.65%)	
Habit of exercise					< 0.001
No	3206 (83.01%)	3139 (81.30%)	3123 (80.78%)	3279 (84.86%)	
Yes	656 (16.99%)	722 (18.70%)	743 (19.22%)	585 (15.14%)	
Smoking status					< 0.001
Never	3204 (82.96%)	2645 (68.51%)	1811 (46.84%)	1367 (35.38%)	
Past	374 (9.68%)	598 (15.49%)	929 (24.03%)	1048 (27.12%)	
Current	284 (7.35%)	618 (16.01%)	1126 (29.13%)	1449 (37.50%)	
Incidence of diabetes					< 0.001
No	3842 (99.48%)	3819 (98.91%)	3791 (98.06%)	3628 (93.89%)	
Yes	20 (0.52%)	42 (1.09%)	75 (1.94%)	236 (6.11%)	

Values are mean ± SD or median (interquartile range) or n (%)

*BMI* Body mass index, *SBP* Systolic blood pressure, *DBP* Diastolic blood pressure, *ALT* Alanine aminotransferase, *AST* Aspartate aminotransferase, *HbA1C* Glycosylated hemoglobin, *TG* Triglyceride, *TC* total cholesterol, *GGT* Gamma-glutamyltransferase, *HDL-C* High-density lipoprotein cholesterol, *FPG* Fasting plasma glucose

### Univariate analysis

Univariate analysis was performed, and the results are shown in Table 2. In summary, age, BMI, SBP, DBP, ALT, AST, TC, TG, FPG, ethanol consumption, past and current smoking status, and GGT/HDL-C were positively associated with the incidence of T2DM. Habitual exercise had no relationship with the incidence of diabetes mellitus. The risk of developing T2DM in males was higher than that in females, and smokers (current and past) also had a higher risk of developing T2DM than never smokers.

**Table 2:** The results of univariate analysis

	Statistics	HR (95% CI)	*P* value
Sex			
Female	7034 (45.52%)	Ref	
Male	8419 (54.48%)	2.53 (1.99, 3.21)	< 0.0001
Age (years)	43.71 ± 8.90	1.06 (1.04, 1.07)	< 0.0001
BMI (kg/m2)	22.12 ± 3.13	1.24 (1.22, 1.27)	< 0.0001
SBP (mmHg)	114.49 ± 14.97	1.03 (1.03, 1.04)	< 0.0001
DBP (mmHg)	71.58 ± 10.50	1.05 (1.04, 1.06)	< 0.0001
ALT (IU/L)	19.99 ± 14.35	1.006 (1.005, 1.007)	< 0.0001
AST (IU/L)	18.40 ± 8.64	1.008 (1.006, 1.010)	< 0.0001
TC (mmol/L)	198.22 ± 33.41	1.010 (1.008, 1.013)	< 0.0001
TG (mmol/L)	80.78 ± 58.07	1.007 (1.006, 1.007)	< 0.0001
HbA1C (mmol/mol)	33.03 ± 3.52	1.44 (1.40, 1.48)	< 0.0001
FPG (mg/dl)	92.96 ± 7.44	1.20 (1.18, 1.22)	< 0.0001
Ethanol consumption (g/week)	47.71 ± 82.31	1.002 (1.001, 1.003)	0.0011
Fatty liver			
No	12,716 (82.288%)	Ref	
Yes	2737 (17.712%)	7.03 (5.71, 8.64)	< 0.0001
Habit of exercise			
No	12,747 (82.49%)	Ref	
Yes	2706 (17.51%)	0.76 (0.56, 1.02)	0.0652
Smoking status			
Never	9027 (58.42%)	Ref	
Past	2949 (19.08%)	1.66 (1.26, 2.19)	0.0003
Current	3477 (22.50%)	2.59 (2.06, 3.25)	< 0.0001
GGT/HDL-C ratio	15.55 ± 15.73	1.02 (1.01, 1.02)	< 0.0001

*CI* Confidence interval, *Ref* Reference

**Fig. 1:**
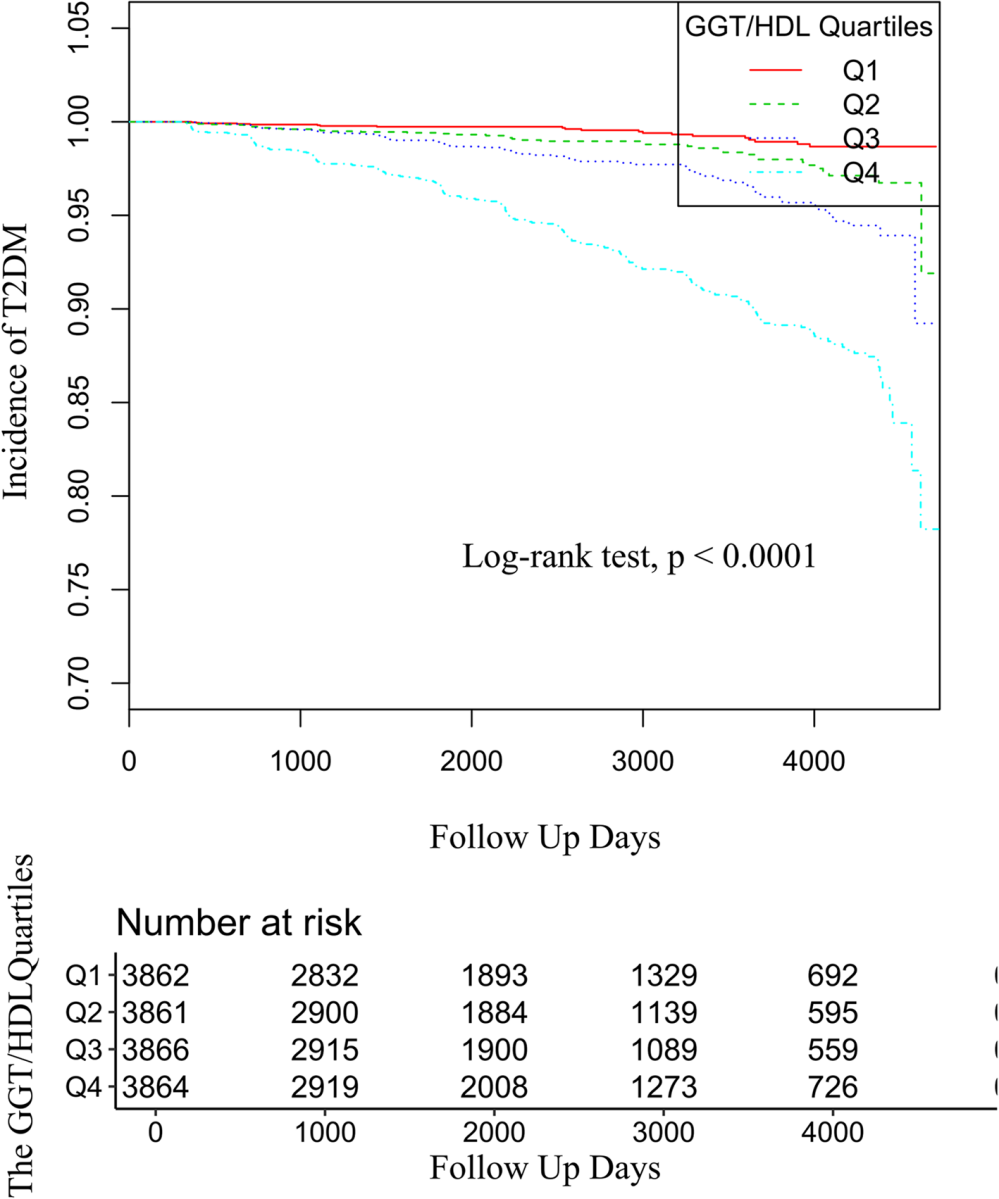
Kaplan–Meier event-free survival curve. Kaplan–Meier event-free survival curve. Kaplan–Meier analysis of the incidence of diabetes based on GGT/HDL-C quartiles (log rank, *P* < 0.0001)

The Kaplan‒Meier curves of the four GGT/HDL-C quartile groups showed that the diabetes incidence risk between each of them was significantly different (the *P* value of the log-rank test < 0.0001). The increase in cumulative diabetes event rates kept pace with the increased GGT/HDL-C quartiles (Fig. 1).

### Relationship between GGT/HDL-C and the incidence of T2DM

In Table 3, we used a Cox proportional hazard regression model to evaluate the associations between GGT/HDL-C and the incidence of T2DM. A nonadjusted model and models with different covariates adjusted are presented. In the crude model and model I (minimally adjusted model), the GGT/HDL-C ratio had a significant positive correlation with the incidence of T2DM: in the crude model, HR = 1.015, 95% CI: 1.013 to 1.017, *P* < 0.00001; in the adjusted model I (adjusted age, sex, and BMI), HR = 1.010, 95% CI: 1.007 to 1.014, *P* < 0.00001. In adjusted model II (fully adjusted model), HR = 1.005, 95% CI: 1.000 to 1.010, *P* = 0.0670; the positive correlation between them was insignificant. The GGT/HDL-C ratio was then handled as a categorical variable (quartile) for the purpose of sensitivity analysis. There was an increased risk for developing T2DM as the quartiles of GGT/HDL-C increased in the crude model and the adjusted model I (both *P* for trend < 0.00001), and we also observed a significant *P* for trend (< 0.00396**)** in the fully adjusted model.

**Table 3:** Relationship between GGT/HDL-C and incident of DM2 in different models

Exposure	Crude model (HR, 95% CI, *P*)	Model I (HR, 95% CI, *P*)	Model II (HR, 95% CI, *P*)
GGT/HDL-C	1.015 (1.013, 1.017) < 0.00001	1.010 (1.007, 1.014) < 0.00001	1.005 (1.000, 1.010) 0.0670
GGT/HDL-C quartiles			
Q1	Ref	Ref	Ref
Q2	2.22 (1.30, 3.78) 0.0033	1.87 (1.09, 3.22) 0.0228	1.28 (0.74, 2.20) 0.3790
Q3	4.04 (2.47, 6.62) < 0.0001	2.75 (1.62, 4.68) 0.0002	1.25 (0.73, 2.16) 0.4171
Q4	11.59 (7.34, 18.30) < 0.0001	6.08 (3.59, 10.29) < 0.0001	1.84 (1.04, 3.24) 0.0361
*P* for trend	< 0.0001	< 0.0001	0.00396

Crude model did not adjust for other covariants

Model I adjusted for age, sex, BMI

Model II adjusted for age, sex, BMI, SBP, DBP, ALT, AST, TC, TG, HbA1C, FPG, fatty liver, smoking and exercise status and ethanol consumption

*CI* Confidence interval, *Ref* Reference

### Nonlinear relationship between GGT/HDL-C and T2DM

**Fig. 2:**
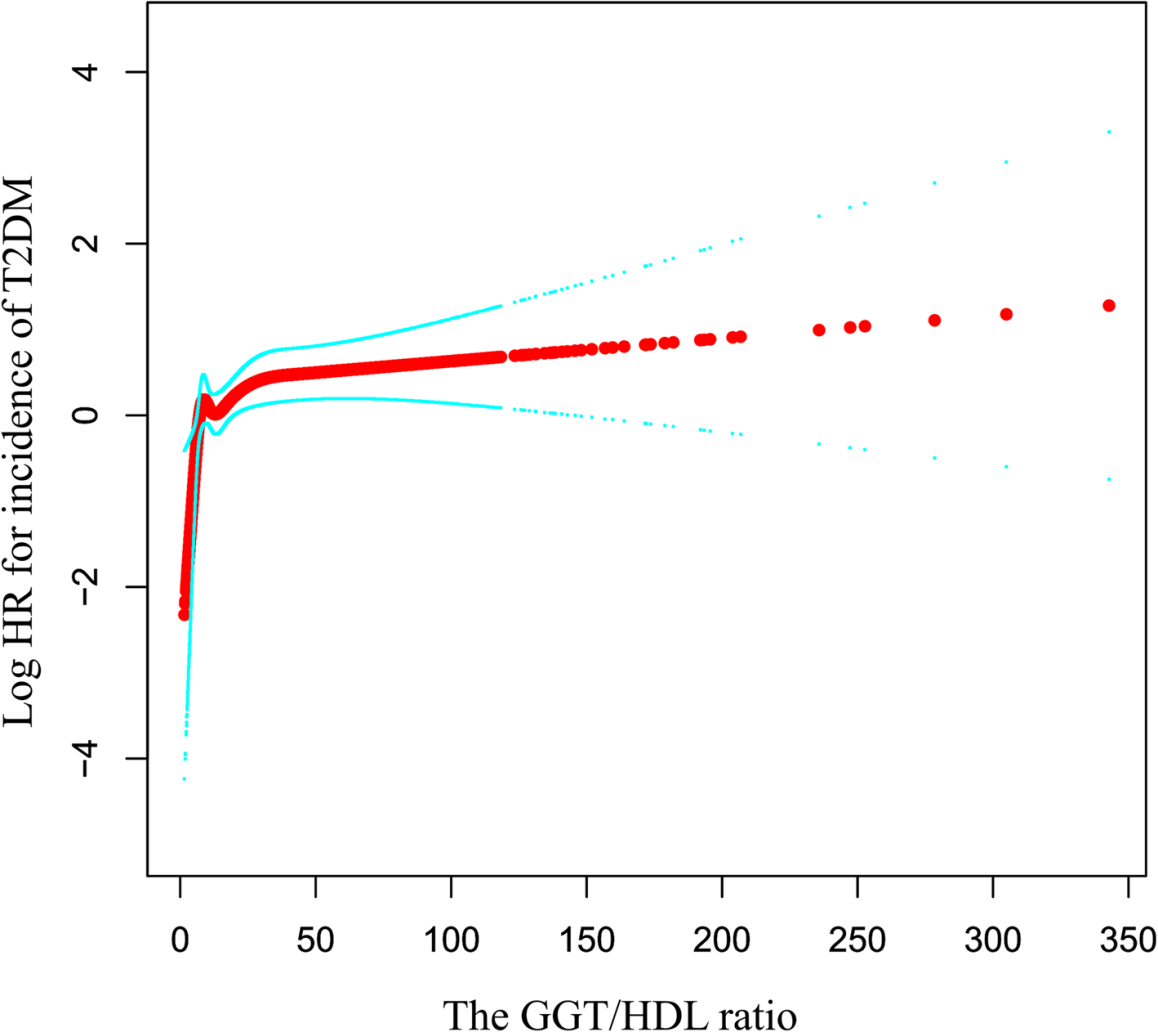
The nonlinear relationship between GGT/HDL-C and the incidence of diabetes

Next, we used GAM to explore whether there was a curvilinear relationship between the independent and dependent variables and showed the results in Fig. 2. We observed a curvilinear relationship after covariates (sex, age, BMI, ALT, AST, TC, TG, HbA1C, FPG, SBP, DBP, smoking, exercise status, ethanol consumption, and fatty liver disease) were adjusted. Subsequent threshold effect analysis found an inflection point in their curvilinear relationship at GGT/HDL equal to 6.35 (log-likelihood ratio test, *P* = 0.001). When GGT/HDL was less than 6.53, the risk of incident T2DM increased with increasing GGT/HDL-C (HR: 2.57, 95% CI: 1.20 to 5.49, *P* = 0.0151). When the GGT/HDL-C ratio was greater than 6.53, the incidence of T2DM no longer increased (HR: 1.00, 95% CI: 1.00 to 1.01, *P* = 0.0803), and their relationship tended to saturate (Table 4).

**Table 4:** The results of the two-piecewise linear regression model

	Incident of diabetes (HR, 95%CI)	*P* value
Fitting model by standard linear regression	1.01 (1.00, 1.01)	0.0670
Fitting model by two-piecewise linear regression		
Inflection point of GGT/HDL-C	6.53	
≤ 6.53	2.57 (1.20, 5.49)	0.0151
≥ 6.53	1.00 (1.00, 1.01)	0.0803
*P* for log likelihood ratio test	0.001	

Adjusted age, sex, BMI, SBP, DBP, FPG, ALT, AST, HbA1C, TC, TG, fatty liver, smoking and drinking status, exercise status

A nonlinear relationship between them was detected after adjusting for age, sex, BMI, SBP, DBP, ALT, AST, TC, TGs, HBA1C, FPG, fatty liver, smoking and exercise status and ethanol consumption.

### Subgroup analysis

We used age, sex, BMI, SBP, DBP, smoking, drinking, exercise status, and fatty liver disease as categorical variables to evaluate the effect sizes in different subgroups and to explore potential interactions in Table 5. Tests for interactions were not statistically significant across all the subgroups listed above (all *P* values for interactions were > 0.05). Considering that the nonlinear relationship between the independent variable and dependent variable might also exist in other subgroups, we supplemented the smoothing plots in stratified age, sex, BMI, smoking, drinking, exercise status, SBP, DBP, and fatty liver disease in Supplemental Fig. 1 (Figure S 1). We discovered that a curvilinear relationship existed in each of the above subgroups. Therefore, the test for interaction needed to consider the existence of the curvilinear relationship and required further in-depth research.

**Table 5:** Effect size of GGT/HDL-C on incident DM2 in prespecified and exploratory subgroups

Character	Number of participant	Effect size (95%CI)	*P* value	*P* for interaction
Age (years)				0.7182
< 60	14,741	1.00 (1.00, 1.01)	0.0903	
> = 60	712	1.00 (0.98, 1.02)	0.9106	
Sex				0.9279
Male	8419	1.005 (0.983, 1.027)	0.6853	
Female	7034	1.006 (1.000, 1.011)	0.0455*	
BMI (kg/m2)				0.6066
< 23	10,059	1.004 (0.996, 1.012)	0.3575	
> = 23	5394	1.006 (1.000, 1.012)	0.0375*	
SBP (mmHg)				0.4241
< 140	14,668	1.00 (1.00, 1.01)	0.1285	
> = 140	785	1.01 (1.00, 1.03)	0.1511	
DBP (mmHg)				0.3525
< 90	14,690	1.00 (1.00, 1.01)	0.1642	
> = 90	763	1.01 (1.00, 1.02)	0.0866	
Current smoker				0.7553
Never	9027	1.00 (0.99, 1.02)	0.3915	
Past	2949	1.01 (1.00, 1.02)	0.1355	
Current	3477	1.00 (0.99, 1.01)	0.4486	
Drinking status				0.5413
Non	11,802	1.00 (1.00, 1.01)	0.2545	
Light	1754	1.00 (0.99, 1.02)	0.5992	
Moderate	1357	1.00 (0.98, 1.01)	0.6772	
Heavy	540	1.02 (1.00, 1.03)	0.0150*	
Fatty liver				0.4586
No	12,716	1.00 (1.00, 1.01)	0.4310	
Yes	2737	1.007 (1.001, 1.014)	0.0290*	
Exercise				0.3589
No	12,747	1.004 (0.998, 1.010)	0.1552	
Yes	2706	1.012 (0.997, 1.027)	0.1164	

Note 1: Above model adjusted for age, sex, BMI, SBP, DBP, FPG, ALT, AST, HBA1C, TC, TG, fatty liver, smoking and drinking status, exercise status

Note 2: In each case, the model is not adjusted for the stratification variable

## Discussion

The role of liver enzymes and lipid profiles in the pathogenesis and progression of T2DM has long been discussed in the literature [4, 19–21]. To the best of our knowledge, this is the first study focusing on the independent relationship between the GGT/HDL-C ratio and the incidence of T2DM. We found that in the Japanese population, GGT/HDL-C was positively associated with the incidence of diabetes, independent of age, sex, BMI, SBP, DBP, FPG, ALT, AST, HbA1C, TC, TGs, fatty liver, smoking status, drinking status, and exercise status. A further nonlinear relationship was explored, and a threshold or a saturation effect was found. The relationship could be characterized as follows: When GGT/HDL-C was less than 6.53, the risk of diabetes increased 1.56 times as the GGT/HDL-C ratio increased by 1 unit, and the risk leveled off when the ratio was greater than 6.53. Further subgroup analysis was conducted, but no interaction was found in the subgroups investigated.

The enzyme GGT is a sensitive index of liver enzymes. Serum GGT can sensitively reflect liver function, and obvious and rapid elevation of the GGT level can be observed in fatty liver (both nonalcoholic and alcoholic), hepatic inflammation, and hepatitis [22]. In addition, elevated serum GGT levels can also be related to various biochemical reactions and various systemic diseases, such as systemic inflammation, oxidative stress, atherosclerosis, metabolic syndrome, and an increased risk of T2DM [23–25]. Since GGT activity is related to oxidative stress, aggravated inflammation, and potential NAFLD, which are major pathological processes involved in T2DM, we speculate that GGT may be involved in the pathogenesis of T2DM [26, 27]. Previous research has already provided considerable evidence of GGT and the incidence of T2DM [6, 28–30].

Several human studies have provided evidence to support that plasma HDL-C levels are inversely correlated with the risk of T2DM [31–33]. Furthermore, rising serum HDL-C levels through extra interventions can be related to remarkable improvement of glycemic regulation in T2DM patients [34]. Dyslipidemia is a known risk factor for T2DM. As one of the major blood lipid components, HDL-C not only undertakes the function of cholesterol retro transportation but also modulates glycometabolism through the improvement of insulin sensitivity and elevation of insulin secretion [34]. Moreover, HDL-C could inhibit oxidative stress and enhance insulin secretion efficiently, which may exert protective effects on the beta cells of the pancreas [35]. In other tissues and organs involved in glucose metabolism, such as skeletal muscle, adipose tissue, and liver tissue, HDL-C has also been found to have the ability to enhance glucose uptake in both insulin-dependent and noninsulin-dependent ways [36]. These findings suggest that abnormal HDL-C levels and dysfunctionality of HDL-C in the human body may give rise to the risk of developing diabetes [37]. Both elevated GGT levels and decreased HDL-C levels can be related to an increased risk of developing T2DM. Therefore, the GGT/HDL-C ratio is posited to have predictive value for T2DM. In a cross-sectional study conducted on Chinese populations, Guofang Feng et al. investigated the independent association between the GGT/HDL-C ratio and NAFLD. They suggested that the function of the ratio was the combination of the two indicators and might exert a predictive value on the prevalence of NAFLD or other related metabolic disorders [7]. A recent cross-sectional study in a Chinese population of T2DM patients also found that in the overweight (BMI greater than 23 kg/m^2^) subgroup, GGT/HDL-C has a positive relationship with NAFLD incidence [38]. Our study is the first to highlight the role of the GGT/HDL-C ratio in T2DM and further confirm a nonlinear relationship. Further and more profound research is needed to reveal the intrinsic mechanism.

### Study strengths and limitations

The following are strong points in our study. First, we use the GAM to clarify that the relationship between the independent and dependent variables is nonlinear, which assists in better understanding the actual correlation between exposures and outcomes. Second, we used rigorous statistical strategies to reduce the impact of a set of confounders on the results, thus minimizing residual confounders. Third, we obtained the positive finding that when the GGT/HDL-C ratio was less than 6.53, the risk of diabetes increased 1.56 times as the GGT/HDL-C ratio increased by 1 unit. The clinical value of this finding is that the positive correlation between GGT/HDL-C and the risk of T2DM could only be observed when GGT/HDL-C is under a certain threshold.

On the other hand, the following limitations must be acknowledged. First, the cohort was conducted by the medical examination program NAGALA in Japan. The data source was the Japanese population, so we could not extrapolate our findings to different ethnic groups or special groups, such as adolescents. Additionally, we could only use available variables in published data and were unable to encompass all variables that may be related to both GGT/HDL-C and T2DM, such as low-density lipoprotein and inflammatory cytokines. In addition, we could only use HDL-C to represent the function of HDLs in our research. Second, T2DM in this cohort study was not diagnosed with a 2-h oral glucose tolerance test, which might lead to some missing cases, and the incidence of diabetes might be lower than they actually were. Third, GGT/HDL-C was only measured at baseline. Its changes over time could not be observed during follow-up days. Finally, residual confounders caused by measurement error in assessing confounders and unmeasured factors could not be completely ruled out. Further research in diverse populations, with more extensive data collection and meticulous research design, is needed.

## Conclusion

The GGT/HDL-C ratio correlated with the incidence of T2DM in a curvilinear form with a threshold effect. Their positive relationship could be observed when GGT/HDL-C was less than 6.53. That is, a one-unit increase in the GGT/HDL-C ratio was associated with a 1.5-fold increase in the risk of incident T2DM. Therefore, the possible curvilinear relationship should be taken into account in the future risk studies of T2DM, and the positive relationship between GGT/HDL and T2DM can only be observed in a specific range of GGT/HDL.

## Supplementary Information

**Additional file 1: Supplemental Figure 1.** The smoothing plots between GGT/HDL-C ratio and T2DM in subgroups. Each plot was adjusted for age, sex, BMI, SBP, DBP, FPG, ALT, AST, HBA1C, TC, TGs, fatty liver, smoking and drinking status, and exercise status, except for the stratification variable.

## Data Availability

The dataset used in this study can be downloaded from the ‘DATADRYAD’ database (www.Datadryad.org).

## Abbreviations

T2DM: Type 2 diabetes mellitus
NAFLD: Nonalcoholic fatty liver disease
BMI: Body mass index
DBP: Diastolic blood pressure
SBP: Systolic blood pressure
ALT: Alanine aminotransferase
AST: Aspartate aminotransferase
GGT: Gamma-glutamyltransferase
HDL: High-density lipoprotein cholesterol
TG: Triglyceride
TC: Total cholesterol
HbA1C: Glycosylated hemoglobin
FPG: Fasting plasma glucose
HR: Hazard ratio
CI: Confidence interval
GAM: General addictive model
