# Supplementary material for: The relationship between the ratio of gamma-glutamyltransferase to high-density lipoprotein cholesterol and the risk of diabetes mellitus using publicly available data: a secondary analysis based on a longitudinal study in Japan
Source: Lipids Health Dis. 2023 Jan 17;22:7. doi: 10.1186/s12944-023-01772-9 (PMC9843936; doi:10.1186/s12944-023-01772-9)

Supplemental Figure 1 The smoothing plots between GGT/HDL-C ratio and T2DM in subgroups. Each plot was adjusted for age, sex, BMI, SBP, DBP, FPG, ALT, AST, HBA1C, TC, TGs, fatty liver, smoking and drinking status, and exercise status, except for the stratification variable.

A


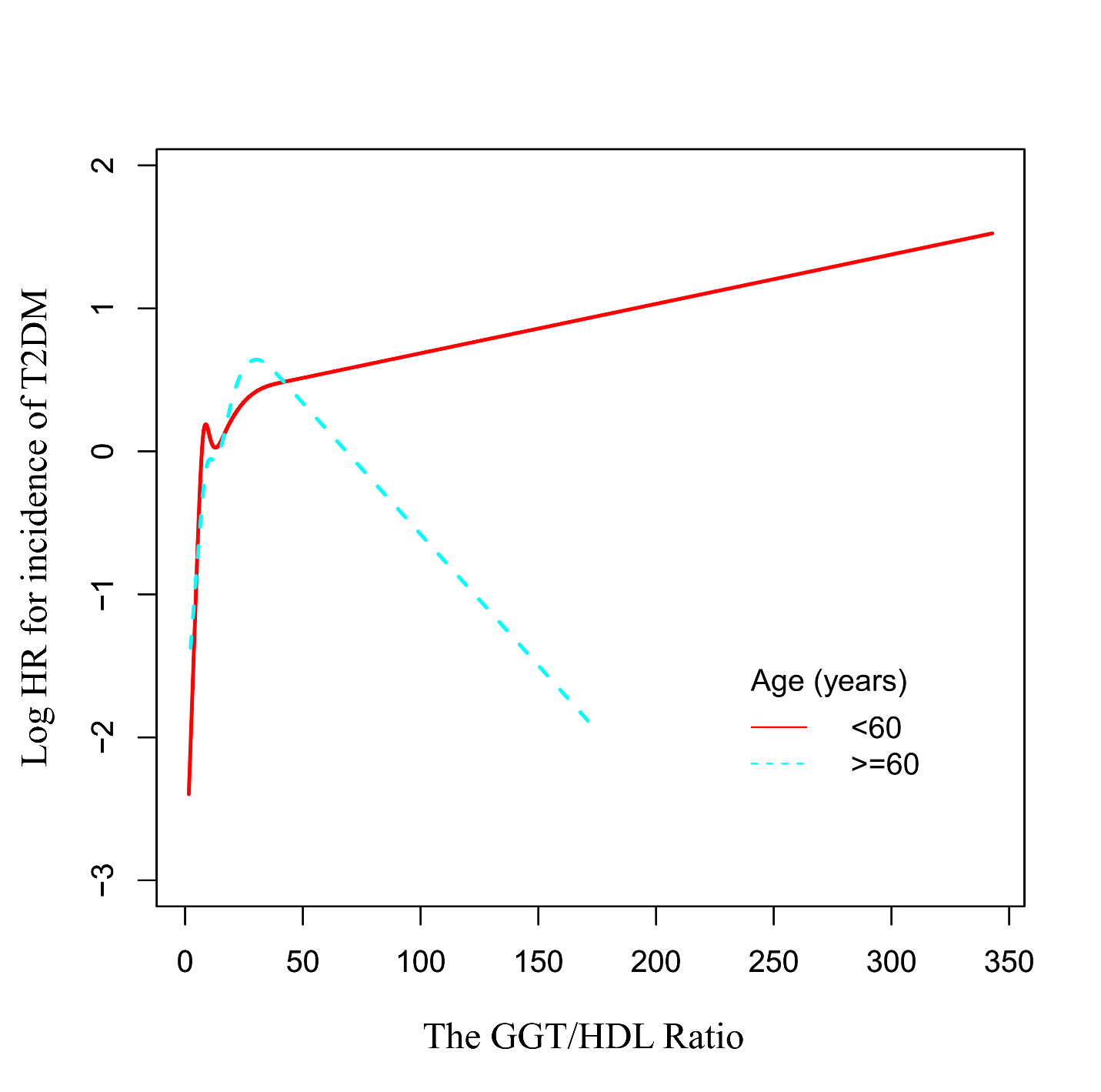


B


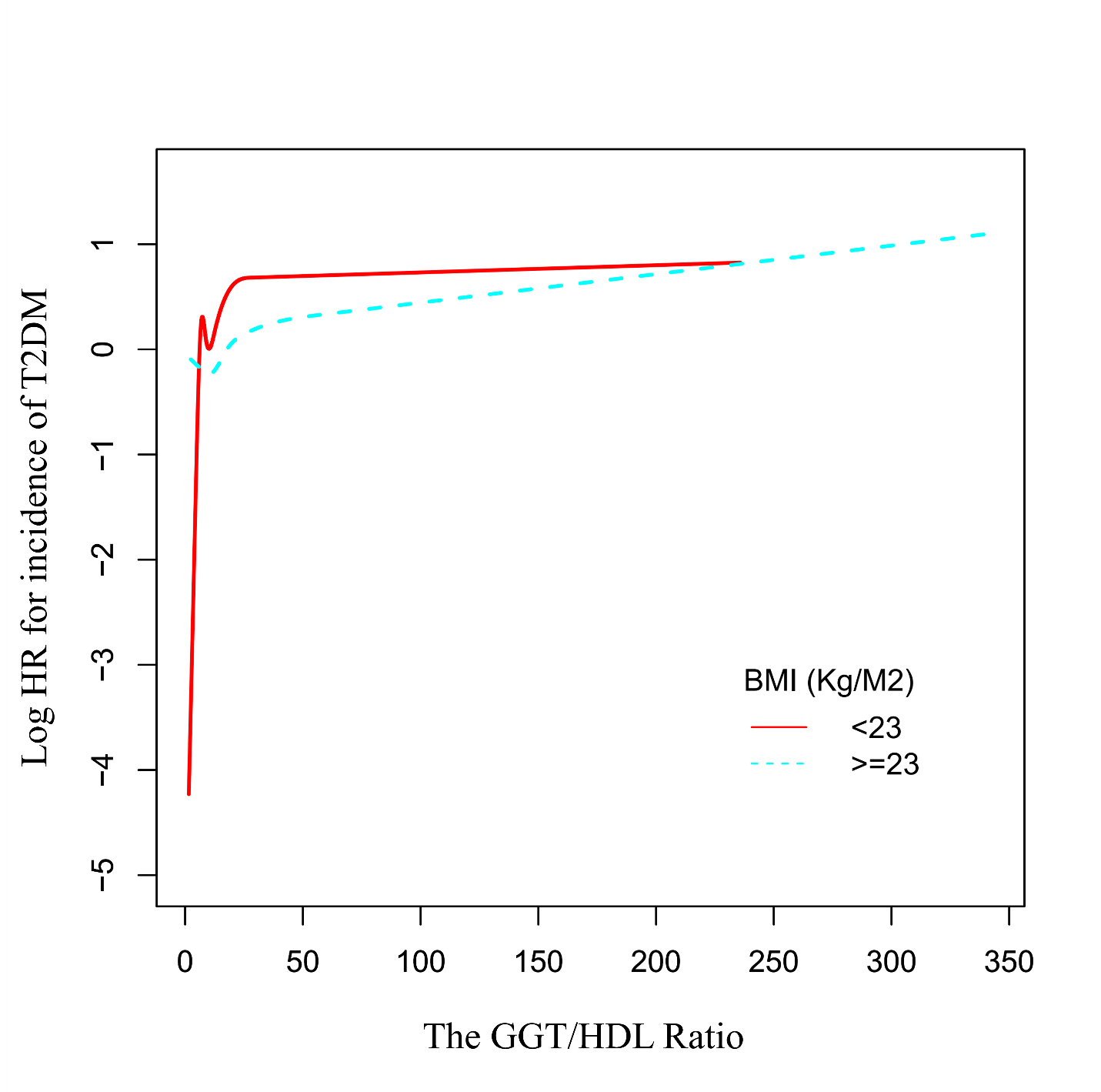


C


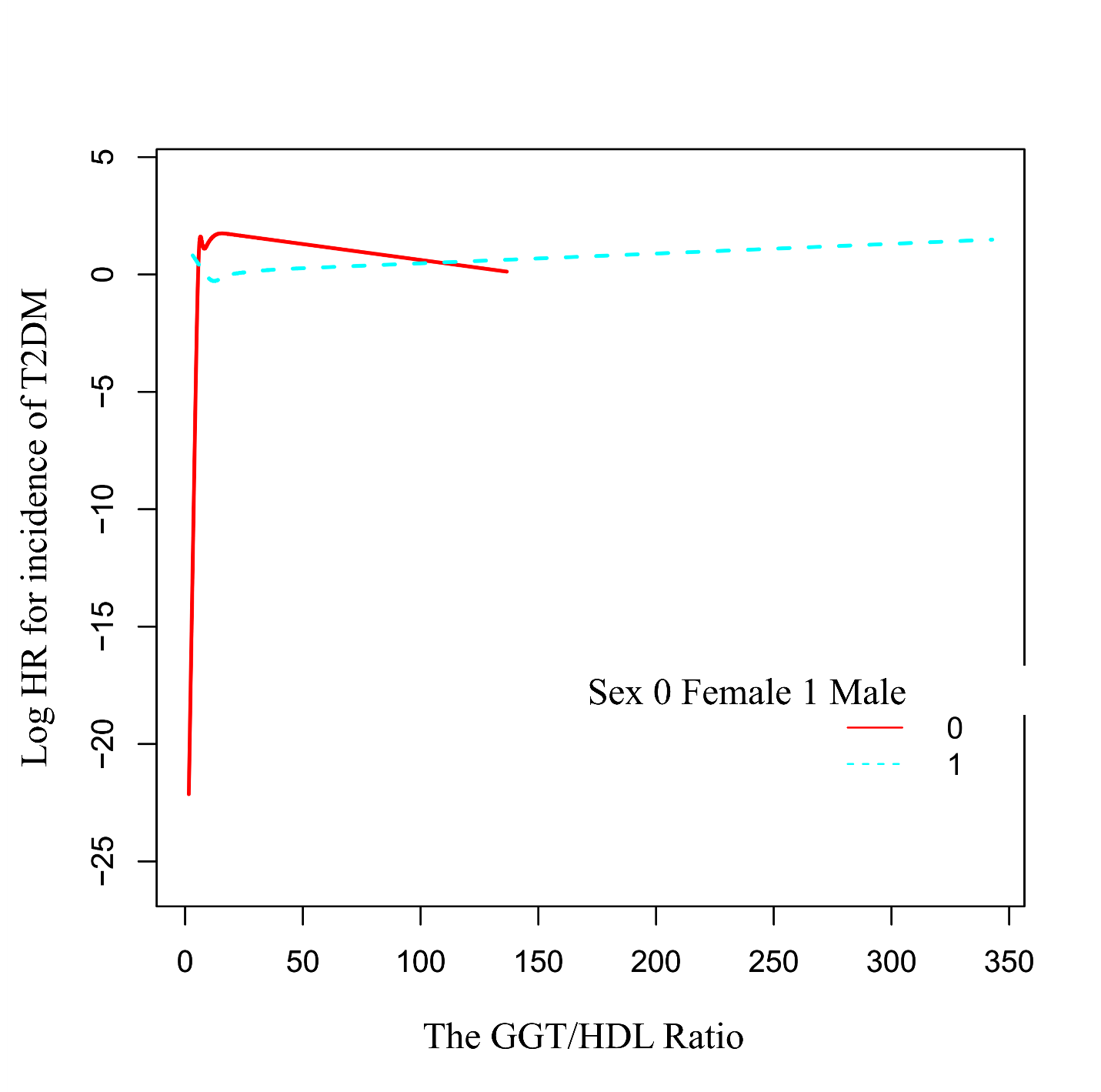


D


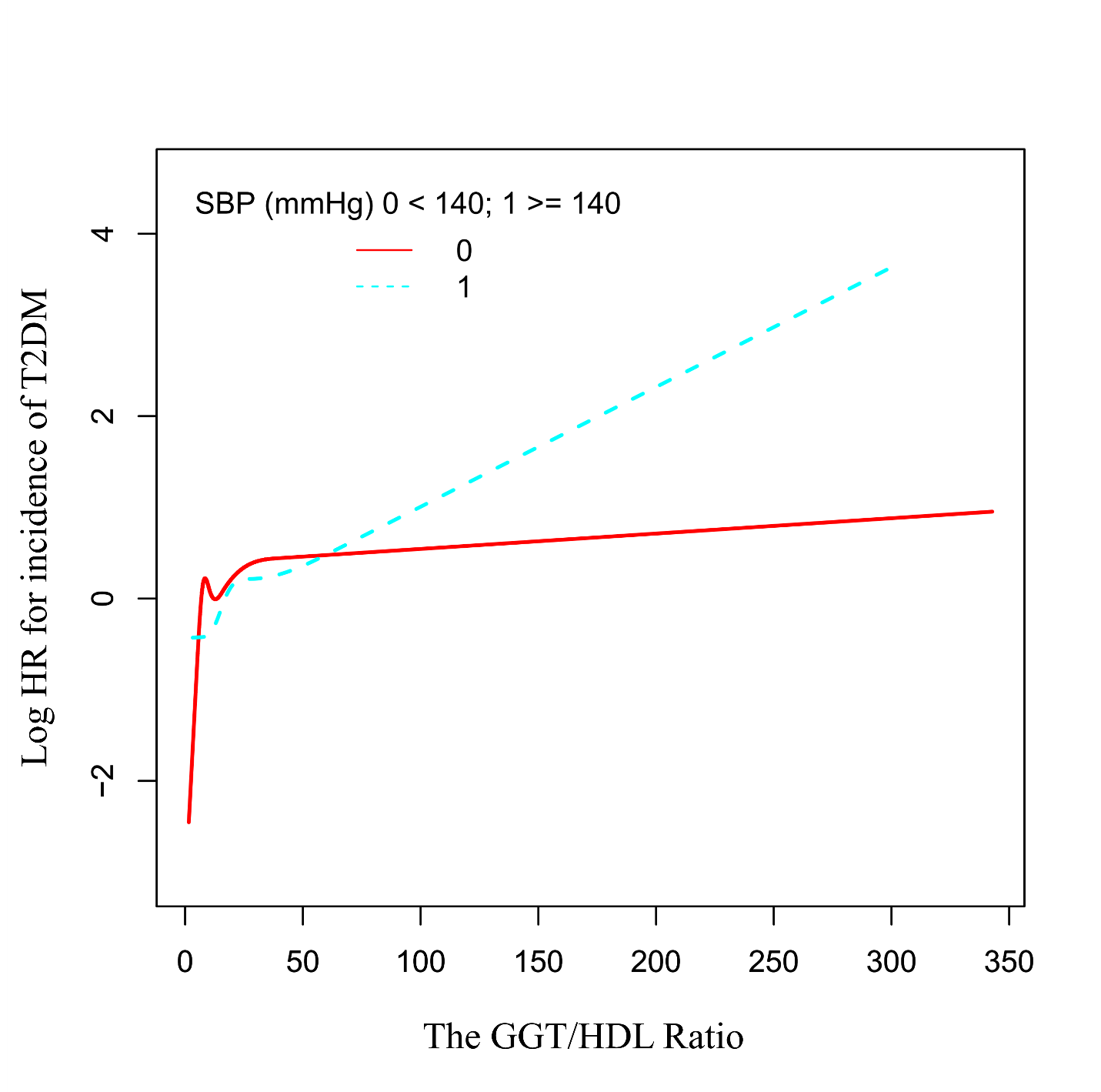


E


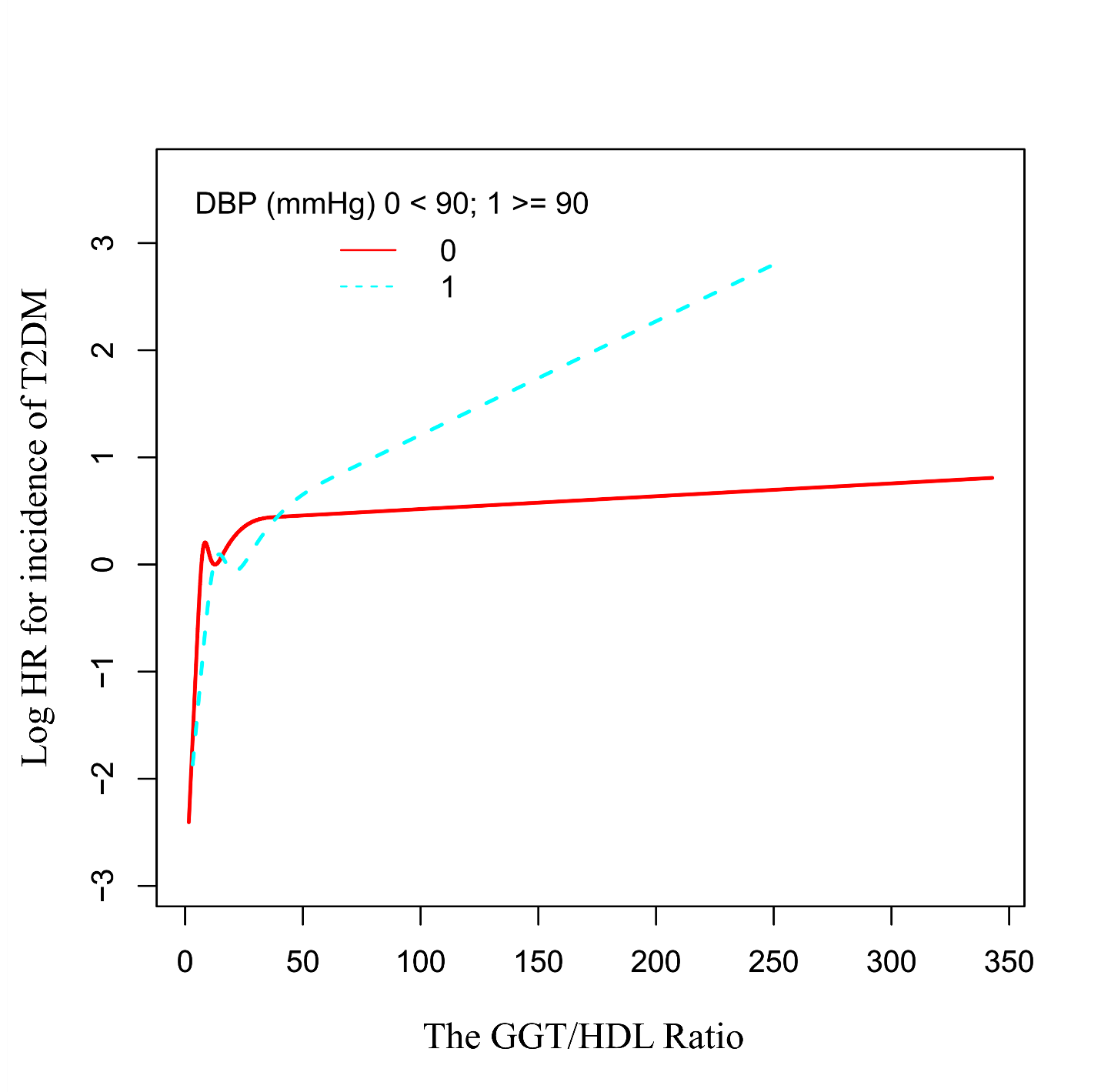


F


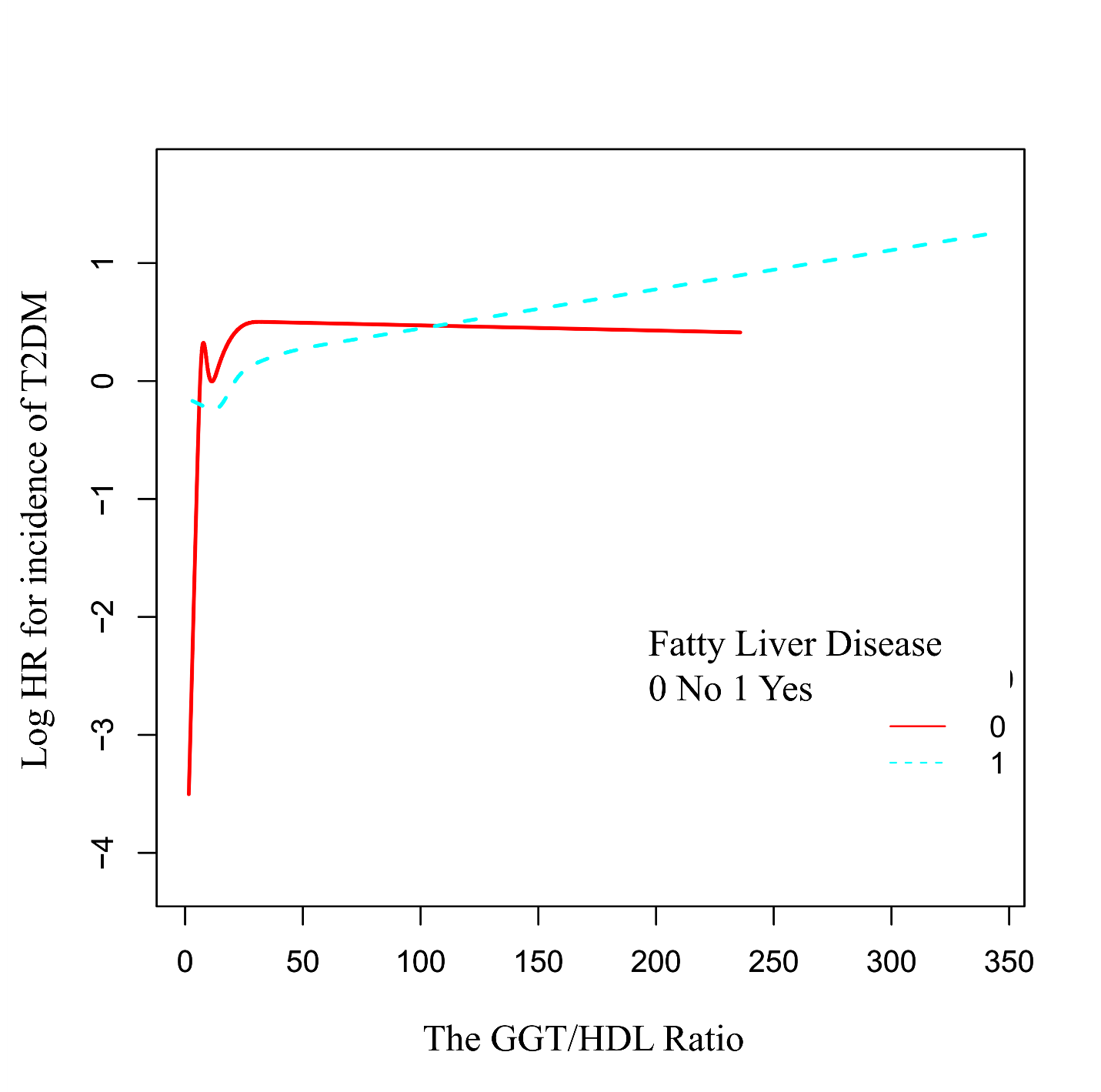


G


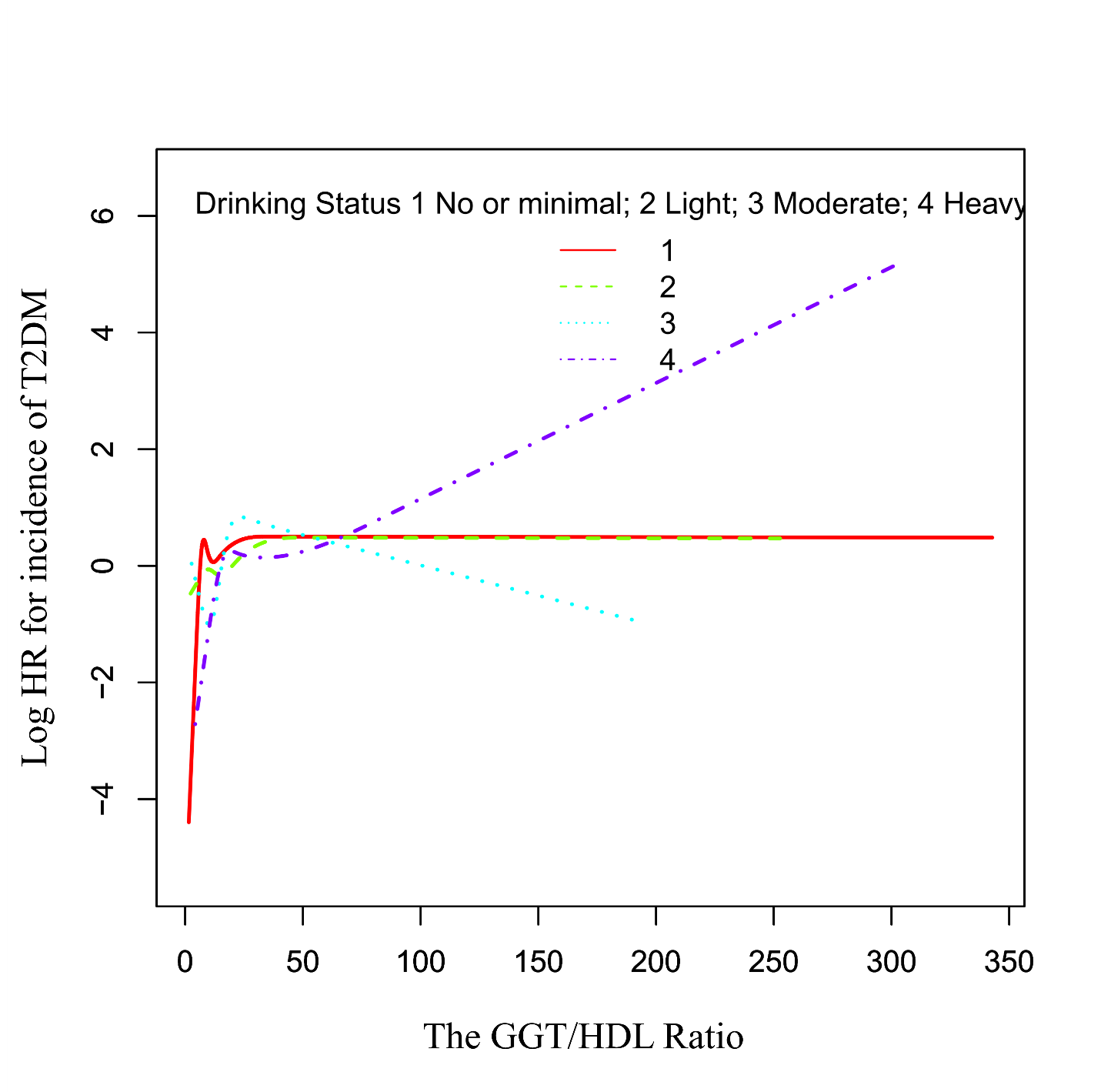


H


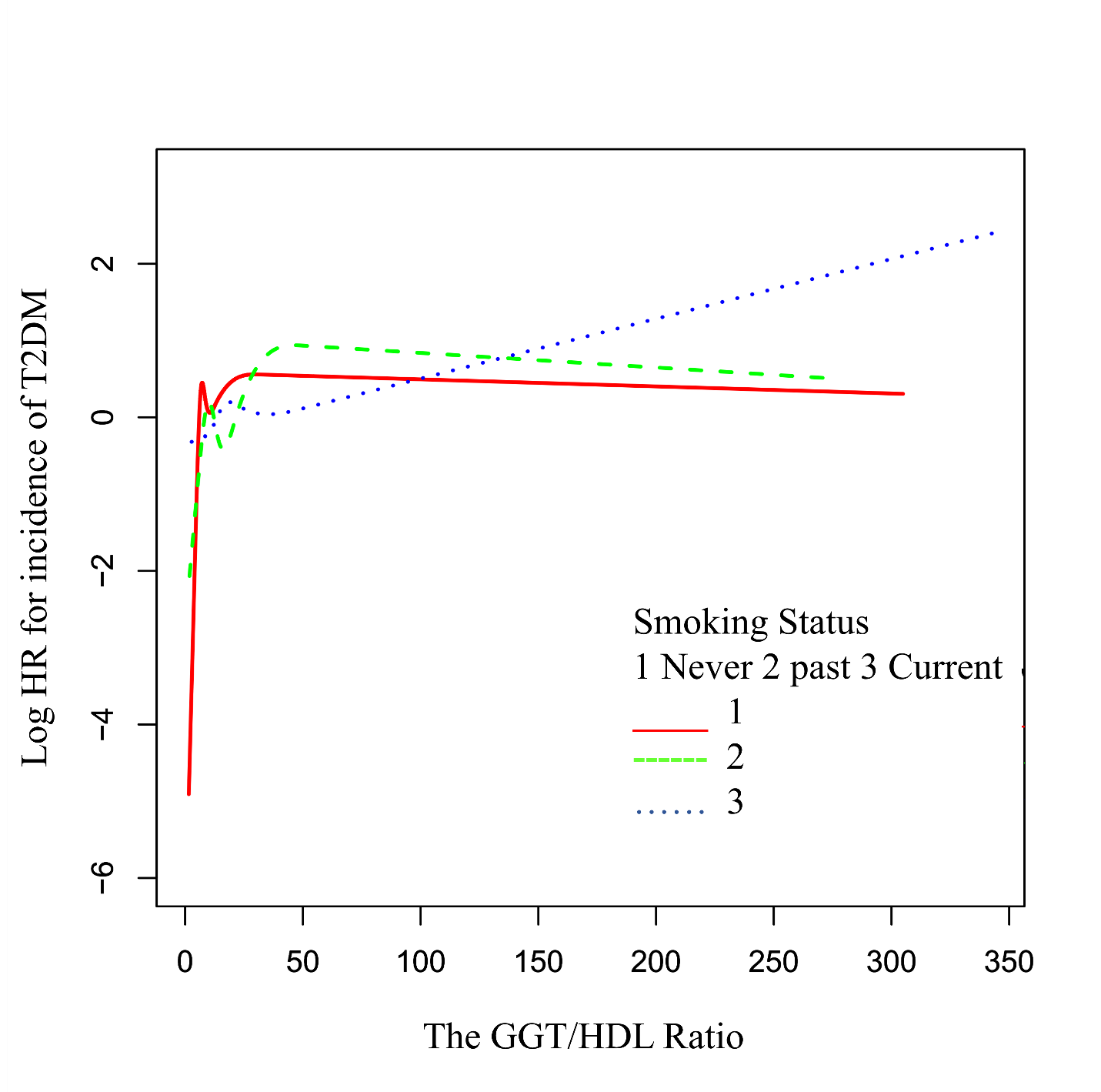


I


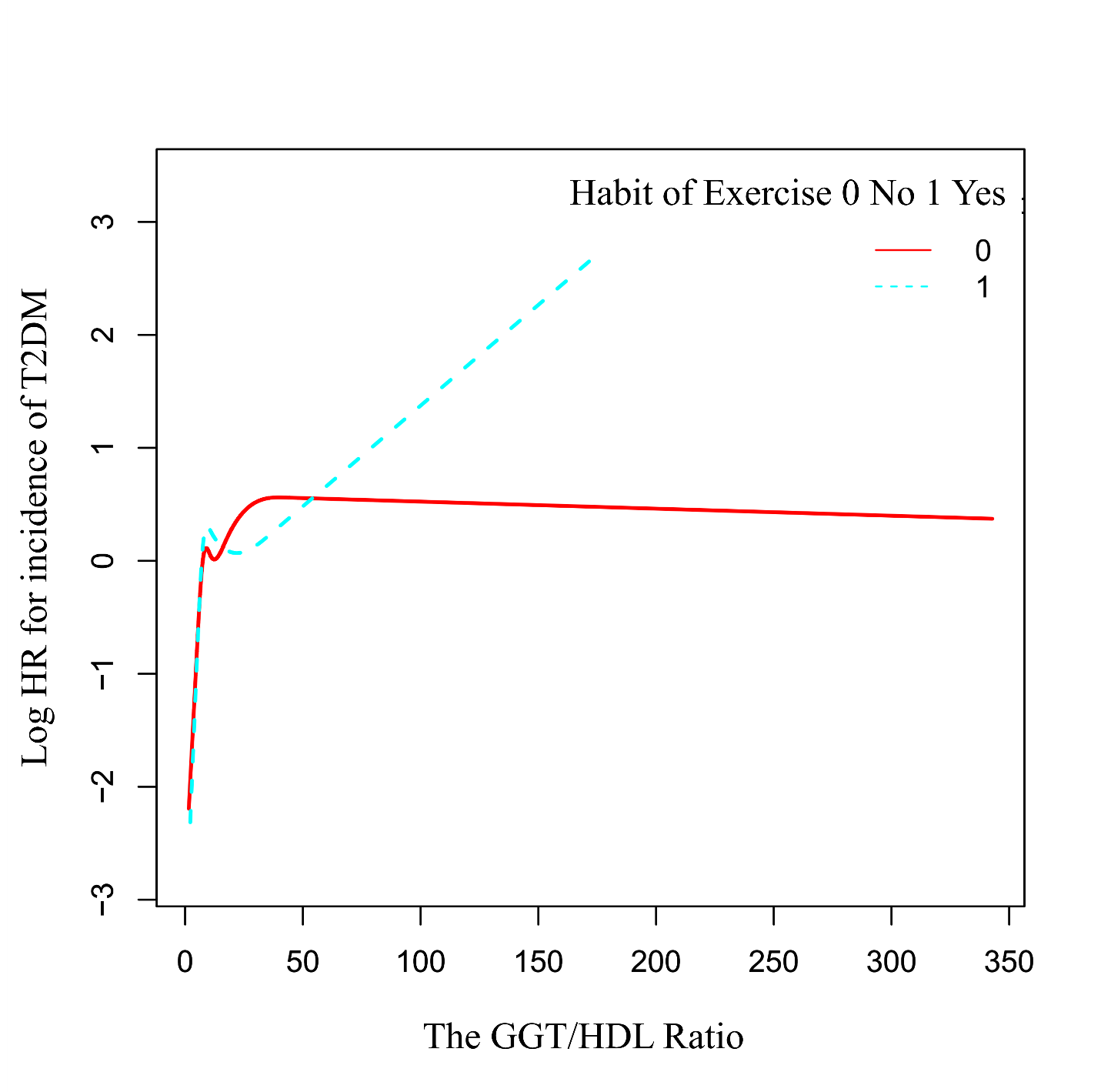

Supplement: Supplementary file 1 — Additional file 1: Supplemental Figure 1. The smoothing plots between GGT/HDL-C ratio and T2DM in subgroups. Each plot was adjusted for age, sex, BMI, SBP, DBP, FPG, ALT, AST, HBA1C, TC, TGs, fatty liver, smoking and drinking status, and exercise status, except for the stratification variable. [file 12944_2023_1772_MOESM1_ESM.docx]
